# The Greenland shark *Somniosus microcephalus*—Hemoglobins and ligand-binding properties

**DOI:** 10.1371/journal.pone.0186181

**Published:** 2017-10-12

**Authors:** Roberta Russo, Daniela Giordano, Gianluca Paredi, Francesco Marchesani, Lisa Milazzo, Giovanna Altomonte, Pietro Del Canale, Stefania Abbruzzetti, Paolo Ascenzi, Guido di Prisco, Cristiano Viappiani, Angela Fago, Stefano Bruno, Giulietta Smulevich, Cinzia Verde

**Affiliations:** 1 Institute of Biosciences and BioResources, CNR, Via Pietro Castellino 111, Naples, Italy; 2 Stazione Zoologica Anton Dohrn, Villa Comunale, Naples, Italy; 3 Dipartimento di Scienze degli Alimenti e del Farmaco, Università di Parma, Parco Area delle Scienze 23/A, Parma, Italy; 4 Dipartimento di Chimica “Ugo Schiff”, Università di Firenze, Via della Lastruccia 3–13, Sesto Fiorentino (FI), Italy; 5 Dipartimento di Biologia, Università Roma 3, Viale Marconi 448, Roma, Italy; 6 Dipartimento di Scienze Matematiche, Fisiche e Informatiche, Università degli Studi di Parma, Parco Area delle Scienze 7A, Parma, Italy; 7 NEST Istituto Nanoscienze, CNR, Piazza San Silvestro 12, Pisa, Italy; 8 Laboratorio Interdipartimentale di Microscopia Elettronica, Università RomaTre, Via della Vasca Navale 79, Roma, Italy; 9 Zoophysiology, Department of Bioscience, Aarhus University, Aarhus, Denmark; James Cook University, AUSTRALIA

## Abstract

A large amount of data is currently available on the adaptive mechanisms of polar bony fish hemoglobins, but structural information on those of cartilaginous species is scarce. This study presents the first characterisation of the hemoglobin system of one of the longest-living vertebrate species (392 ± 120 years), the Arctic shark *Somniosus microcephalus*. Three major hemoglobins are found in its red blood cells and are made of two copies of the same α globin combined with two copies of three very similar β subunits. The three hemoglobins show very similar oxygenation and carbonylation properties, which are unaffected by urea, a very important compound in marine elasmobranch physiology. They display identical electronic absorption and resonance Raman spectra, indicating that their heme-pocket structures are identical or highly similar. The quaternary transition equilibrium between the relaxed (R) and the tense (T) states is more dependent on physiological allosteric effectors than in human hemoglobin, as also demonstrated in polar teleost hemoglobins. Similar to other cartilaginous fishes, we found no evidence for functional differentiation among the three isoforms. The very similar ligand-binding properties suggest that regulatory control of O_2_ transport may be at the cellular level and that it may involve changes in the cellular concentrations of allosteric effectors and/or variations of other systemic factors. The hemoglobins of this polar shark have evolved adaptive decreases in O_2_ affinity in comparison to temperate sharks.

## Introduction

Organisms living in polar environments are exposed to strong environmental constraints. Temperature is often the main environmental stress or adaptation driver over timescales ranging from hours to millennia.

Arctic regions are inhabited by cold-adapted species, which may be either stenothermal or eurythermal, likely depending on ambient temperature variability and evolutionary history [1, 2]. Eurythermal species predominate, because the continuous coastlines from tropical to polar latitudes increase the opportunities for north-south migrations. In contrast, the constant water temperature in the Southern Ocean, where fluctuations rarely exceed 2°C, favoured the evolution of stenothermal animal life [3, 4].

In the Antarctic sea, the modern chondrichthyan genera are scarcely represented, probably as an ecological consequence of specific peculiar trophic or habitat conditions in the Southern Ocean. In contrast, in the Arctic, cartilaginous fishes, such as sharks and skates, are notably present, with about 8% of the species [5].

Greenland sharks typically inhabit deep and extremely cold waters, although distribution is quite wide [6]. *Somniosus microcephalus* is the largest fish species in the Arctic Ocean and likely plays an important ecological role in the marine ecosystem. In fact, widespread changes in the Arctic ecosystem, as a consequence of climate, have led to an increased attention on trophic dynamics and on the role of potential apex predators such as *S*. *microcephalus* in the structure of Arctic marine food webs [6]. Although *S*. *microcephalus* is an Arctic species *sensu stricto*, thriving in areas seasonally covered by sea ice, it is also known to enter more temperate waters in the North Atlantic [7, 8]. Recent results demonstrated that *S*. *microcephalus* is one of the longest-living vertebrate species, with the largest captured animal (502 cm) estimated to be almost 400 years old [9]. This finding is in agreement with observations that elasmobranchs are among the most long-living vertebrates [10]. The remarkable evolutionary success of elasmobranchs has raised considerable interest in their respiratory control mechanisms [11]. However the hemoglobins (Hbs) of polar cartilaginous fish have been studied less extensively than those of polar teleosts. One difficulty that presumably hindered investigating their structure–function relationships is the presence of multiple isoforms [12–17].

Erythrocytes are notably larger than those of most vertebrates, including teleosts [18]. Large erythrocytes might limit the efficiency of the O_2_-transport system through both the dynamics of gas transfer across a large erythrocyte, and the flow properties of the blood [19]. In migratory fish, evolutionary adaptations or physiological compensations, to adjust O_2_ demand (metabolism) and supply (O_2_ uptake and transport) to ambient O_2_, have often been observed and well documented, and the ability of fish to colonise a wide range of habitats has often evolved together with the molecular and functional modulation of the Hb system [20–23].

The present study was undertaken to characterise the structural and functional properties of the Hb of *S*. *microcephalus*, one of the longest-living vertebrate species. The specific aims were (i) to characterise the ligand properties and O_2_ binding of these Hbs and their modulation by red blood cell physiological effectors; (ii) to structurally characterise the Hbs by using a combination of spectroscopic techniques for gaining information on the heme cavity, with regard to heme oxidation and coordination states.

## Materials and methods

### Collection of specimens

During the TUNU-V expedition in August 2013, specimens of the Greenland shark *S*. *microcephalus* were caught by long lines, set at 700 m or more, in Tyrolerfjord, NE Greenland (74N, 21W). All procedures were conducted in accordance with the Animal Welfare Act and were approved by the Arctic University of Norway, Norway. The capture of sharks was carried out in strict accordance with laws and regulations and with authorisation from the Government of Greenland (Ministry of Fisheries, Hunting & Agriculture, document number 935119). Blood was withdrawn from two individuals (220 and 320 cm total length) with heparinised syringes from a major vein in the back of the head soon after capture. We have performed the purification of Hbs in the blood taken from both specimens, as well as in other samples taken in TUNU-VI (2015), always obtaining identical results. Therefore, on these grounds, the whole study on Hb characterisation was performed on a single specimen.

Saline-washed erythrocytes were immediately frozen and stored at −80°C. Hemolysates were prepared as described previously [24].

### Hb purification

Hb purification was achieved by anion-exchange chromatography at 4°C on a Mono Q-Tricorn column (1.0 ×10 cm) mounted on an AKTA-FPLC system (Pharmacia, Uppsala, Sweden). The column was equilibrated with 20 mM Tris-HCl, pH 8.0 (buffer A); Hb elution was performed with a gradient from buffer A to buffer B (80 mM NaCl, pH 8.0, in buffer A). The major Hbs, namely Hb 1, Hb 2 and Hb 3, were eluted at 25, 35 and 50% buffer B, respectively. To improve the separation, the hemolysate was treated with 10 mM dithiotreitol (DTT) overnight at 4°C under anaerobic conditions. Hb multiplicity and purity were analysed by isoelectrofocusing (IEF, pH 3.0 to 9.0) on polyacrylamide gels (Phastgel, GE Healthcare Biosciences AB, Uppsala, Sweden). Hbs were saturated with CO and stored at −20°C.

The *S*. *microcephalus* Hb concentration was estimated spectrophotometrically at 540 and 569 nm, using the millimolar extinction coefficient of the ferrous carbonylated-heme (ε = 13.4 mM^−1^cm^−1^) [25].

### Protein sequence analysis

*S*. *microcephalus* globins were purified by reverse-phase HPLC on a micro-Bondapak-C18 (0.39 cm × 30 cm) column equilibrated with 25% acetonitrile (CH_3_CN) and 0.3% trifluoracetic acid (TFA) (solvent A). Elution was performed with a gradient of 90% CH_3_CN and 0.1% TFA (solvent B) in solvent A; the absorbance was monitored at 280 nm. Before loading, samples were incubated in a denaturating solution of 20% β-mercaptoethanol and 4% TFA at room temperature. The purified globins (2 μg) were reduced with 1% DTT (15 minutes at 50°C), alkylated with 4% iodoacetamide (15 minutes) and separately digested with trypsin, thermolysin A, pepsin (Sigma-Aldrich) and proteinase K (Promega) using the digestion protocols recommended by the manufactures. Mass spectrometry was carried out using a LTQ-Orbitrap coupled with a Phenomenex Aeris™ PEPTIDE 3.6 μm XB-C18 (0.21 cm × 15 cm) reverse-phase column developed in a 0.2% formic acid/water-0.2% formic acid/CH_3_CN gradient. The flux was 200 μl/min. A precursor mass tolerance of 10 ppm and a fragment mass error tolerance of 0.05 Da were applied. The analysis of all peptide mixtures was carried out using the software PEAKS Studio (version 7.5, Bioinformatics Solutions, Waterloo, Canada). The sequences of the α and β chains of *Squalus acanthias* Hb [26] were used as starting templates.

### UV-Vis and resonance raman spectroscopy

Ferric *S*. *microcephalus* Hbs at pH 5.0, 7.6, and 10.6 were prepared in 50 mM MES [2-(N-morpholino)ethanesulfonic acid], 20 mM Tris-HCl and 50 mM glycine, respectively. The hydroxyl complex in isotopically enriched water was prepared by equilibrating Hbs in 20 mM Tris-HCl pH 7.6 with 0.1 mM glycine pH 11.0 prepared with D_2_O (99.8%; purchased from Merck AG Darmstadt, Germany). Ferrous samples at pH 7.6 were prepared by addition of a freshly prepared sodium dithionite solution (10 mg/mL) to the ferric forms previously degassed with nitrogen. The Fe(II)-CO complexes at pH 7.6 were prepared by flushing ferric Hbs firstly with nitrogen, then with CO and reducing the heme by addition of a freshly prepared sodium dithionite solution (10 mg/mL). Isotopically enriched gaseous CO was purchased from Rivoira (Milan, Italy). The Fe(II)-O_2_ complexes at pH 7.6 were prepared by reduction of the ferric form with a freshly prepared sodium dithionite solution (10 mg/mL), followed by gel filtration on a Sephadex G-25 Medium column equilibrated with the Tris-HCl buffer. Protein concentrations in the range 5–30 μM were used. The protein concentration was estimated on the basis of the molar absorptivity, ε = 150 mM^-1^ cm^-1^ at 405 nm.

The resonance Raman (RR) spectra obtained with a laser excitation wavelength in resonance with the Soret band leads to the intensification of the heme vibrational modes. Therefore, the heme active site can be probed and information on the structure–function relationship obtained. In the high-frequency region of the RR spectrum, the skeletal modes, called core-size marker bands, are very sensitive to both porphyrin geometry and electronic structure. Their frequency is inversely correlated with the size of the core, and, therefore, depends on the coordination and spin states of the heme iron atom [27]. Moreover, in the low-frequency region of the RR spectrum, the Fe–ligand stretching vibrations (Fe-OH^-^, Fe-CO, Fe-N_His_) can be detected. Since their frequency depends on the interaction between the ligand and the surrounding amino-acid residues, information on the role of the distal and proximal residues in ligand stabilisation and interactions can be obtained [28].

The combination of the complementary techniques electronic absorption and RR spectroscopy has been extensively applied to heme proteins, since they provide specific structural information with regard to heme oxidation, coordination, and spin states as well as detailed information on the nature of the ligands of the heme Fe [28]. In particular, the electronic absorption spectrum is characterised by the π→π* electronic transitions in the Soret (380–440 nm) and visible Q band (500–600 nm) regions deriving from the heme group [29] and, in the region between 600 and 650 nm, by charge transfer bands typical only of high-spin species [30].

Electronic absorption measurements of *S*. *microcephalus* Hbs were recorded using a 5-mm Nuclear Magnetic Resonance (NMR) tube (300-nm/min scan rate) or a 1-cm cuvette (600-nm/min scan rate) at 25°C in a Cary 60 spectrophotometer (Agilent Technologies) with a resolution of 1.5 nm. Absorption spectra were measured both prior and after RR measurements to ensure that no degradation occurred under the experimental conditions used.

RR spectra were obtained at 25°C using a 5-mm NMR tube by excitation with the 406.7 and 413.1 nm lines of a Kr^+^ laser (Innova 300 C, Coherent, Santa Clara, CA, USA) and the 441.6 nm line of a He–Cd laser (Kimmon IK4121R-G). Backscattered light from a slowly rotating NMR tube was collected and focused into a triple spectrometer (consisting of two Acton Research SpectraPro 2300i and a SpectraPro 2500i in the final stage with a 3600-grooves/mm or 1800-grooves/mm grating) working in the subtractive mode, equipped with a liquid nitrogen-cooled CCD detector. A spectral resolution of 1.2 cm^−1^ and spectral dispersion of 0.4 cm^−1^/pixel were calculated theoretically on the basis of the optical properties of the spectrometer for the 3600 grating; for the 1800 grating, used to collect the RR spectra of the Fe(II)-CO complexes in the 2000–2300 cm^−1^ region, the spectral resolution was 4 cm^−1^ and spectral dispersion 1.2 cm^−1^/pixel. A cylindrical lens, which focuses the laser beam in the sample to a narrow strip rather than the usual point, was used to collect the spectra of the Fe(II)-CO and Fe(II)-O_2_ complexes in order to avoid photolysis. RR spectra obtained with a laser excitation wavelength in resonance with the Soret band leads to the intensification of the heme vibrational modes. Therefore, the heme active site can be probed and information on the structure–function relationship obtained. In the high-frequency region of the RR spectrum, the skeletal modes, called core-size marker bands, are very sensitive to both porphyrin geometry and electronic structure. Their frequency is inversely correlated with the size of the core, and, therefore, depends on the coordination and spin states of the heme iron atom [27]. Moreover, in the low-frequency region of the RR spectrum, the Fe–ligand stretching vibrations (Fe-OH^-^, Fe-CO, Fe-N_His_) can be detected. Since their frequency depends on the interaction between the ligand and the surrounding amino-acid residues, information on the role of the distal and proximal residues in ligand stabilisation and interactions can be obtained [28].

The RR spectra were calibrated with indene, n-pentane and carbon tetrachloride as standards to an accuracy of 1 cm^−1^ for intense isolated bands. All RR measurements were repeated several times under the same conditions to ensure reproducibility. To improve the signal-to-noise ratio, a number of spectra were accumulated and summed only if no spectral differences were noted. All spectra were baseline-corrected.

### O_2_-equilibrium measurements

O_2_-binding equilibrium curves were measured at 15 and 25°C. The temperature of 15°C was chosen because *S*. *microcephalus*, although it shows a marked preference for cold water (from −1.8 to 10°C) [7, 31], is also known to enter more temperate waters in the North Atlantic [6]. The temperature of 25°C was selected to make a comparison with parameters measured for Hb of *Mustelus griseus* [32] and *Dasyatis akajei* [17, 33]. For technical limitations of the instrumentation, the O_2_-binding equilibrium curves could not be measured at 0°C. However, the *P*_*50*_ (the *P*_O2_ at which Hb is half-saturated with O_2_) values of *S*. *microcephalus* Hbs at 0°C and at pH 7.8 could be calculated considering linear vant’Hoff and Bohr plots in Hbs (within the examined temperatures and pH ranges), including shark Hbs [17]. O_2_-binding equilibrium curves were measured on 5-μl samples of ion-exchange purified Hb (i.e. cofactor free or ‘stripped’) in 0.1 M Tris-HCl, 0.5 mM EDTA, 0.1 M KCl, using a thin-layer modified gas-diffusion chamber [34–36] connected to cascaded Wösthoff gas-mixing pumps for mixing pure N_2_ with O_2_ or air, as previously described [37–41]. The *P*_O2_ inside the chamber was changed stepwise and the resulting changes in absorbance were continuously measured at 436 nm to monitor that sufficient equilibration had taken place at each step (typically 3–5 min). For each curve, 4–6 saturation steps were measured in the range ~20–80%. To assess the effect of the red blood cell cofactor ATP on O_2_ affinity and cooperativity, experiments were performed in the absence and presence of 2 mM ATP, i.e. at saturating concentrations of the erythrocyte organophosphate cofactor; the Hb concentration was 0.2 mM (on a heme basis). For each O_2_-equilibrium curve, values of *P*_50_ and of the Hill coefficient *n*_*H*_ were obtained from fitting the sigmoidal Hill equation *Y* = *P*_O2_^*n*^/(*P*_50_^*n*^+ *P*_O2_^*n*^) to 4–6 saturation steps, where *Y* is the fractional saturation and *P*_O2_ is the O_2_ partial pressure. Total Cl^−^ concentration and pH of Hb solutions were measured using a 926S Mark II chloride analyser (Sherwood Scientific Ldt, Cambridge, UK) and in Lab micro pH-electrode, respectively, both equipped with a Seven Compact pH/Ion Meter S220 (Mettler Toledo‎, Greifensee, Switzerland), after equilibration of the Hb samples at 15 and 25°C in a HLC BioTech block thermostat Model TK23 (Bovenden, Germany).

The effect of urea on O_2_ binding was investigated at 20°C, using a cuvette fused to a gas-tight reservoir [42, 43]. Briefly, solutions containing 30 μM Hb, 0.1 M KCl, 0.1 M potassium phosphate, 1 mM EDTA, 2.0 mM ATP, at pH 7.4, in the absence and presence of 133 mM urea [44], were equilibrated with humidified O_2_/He mixtures generated using an Environics 4000 gas mixer (Environics Inc., Tolland, CT, U.S.A.), with the O_2_ partial pressure ranging between 0 and 760 Torr. Spectra in the 380- to 500-nm range were recorded with a Cary 4000 spectrophotometer (Agilent Technologies, Lexington, MA, U.S.A.).

Data were fitted to a linear combination of reference spectra, i.e. the spectra of deoxygenated, oxygenated, and oxidised Hb, plus a baseline correction consisting of a horizontal offset, using the software Sigmaplot (Systat Software, Inc.). Briefly, the spectrum of the fully oxygenated species was obtained by exposing the protein solution to pure oxygen; the spectrum of the deoxygenated form was obtained by equilibrating the protein solution with pure helium followed by addition of 1 mM sodium dithionite; the spectrum of the met-Hb form was collected upon addition of 0.1 mM sodium hexacyanoferrate(III) [42].

### Enthalpy change

The overall enthalpy of oxygenation (*ΔH*, kcal mol^−1^; 1 kcal = 4.184 kJ), corrected for the heat of O_2_ solubilisation (−3 kcal mol^−1^), was calculated from the *P*_50_ values measured at 15 and 25°C by using the van’t Hoff equation [45]:

$$\Delta H=-4.574\times [\left(T_{1}\times T_{2}\right)/(T_{1}-T_{2})]\times \Delta \log P_{50}/1000$$

where *T*_1_ and *T*_2_ are the absolute temperatures (in degrees K) corresponding to 15 and 25°C.

### Determination of k_off,R_ for O_2_

*S*. *microcephalus* Hb 1, Hb 2 and Hb 3 were adjusted to 20 μM in a solution containing 0.1 M Tris-HCl, 0.5 mM EDTA, 0.1 M KCl at pH 6.5, in the absence or presence of 2 mM ATP. The solutions were then fluxed with pure nitrogen for 5 minutes to remove unbound O_2_. The samples were then loaded on a stopped-flow apparatus (Applied Photophysics) and mixed with the same CO-equilibrated solution. The displacement of O_2_ by CO was monitored at 420 nm and the time courses were analysed as mono-exponential decays. The value of *k*_off,R_ (O_2_ dissociation constant of Hb in the R state) is estimated as the reciprocal of the lifetime in the exponential decay. Considering that Hbs at the beginning of the displacement reaction were fully oxygenated, the resulting apparent *k*_off_s are referred to the R state. All experiments were carried out at 25°C. For each condition, at least two independent experiments were carried out.

### CO-rebinding kinetics

In laser flash photolysis, a nanosecond laser pulse is used to break the chemical bond between the gaseous ligand (in this work CO) and the Fe atom of the heme. The photodissociated ligand can be rebound on the nanosecond-to-microsecond time scale from temporary docking sites, located inside the protein matrix, to give the so called geminate recombination, or escape to the solvent. A diffusion-mediated, bimolecular rebinding reaction brings the system back, on the micro-to-millisecond time scale, to the pre-perturbation equilibrium. When ligation is coupled to conformational transitions, photodissociation also triggers tertiary and, for multisubunit proteins, quaternary relaxations that result in changes in ligand-binding rates. In Hbs, the quaternary relaxation usually occurs before bimolecular rebinding to the fast-rebiding R state is completed, thus it is possible to observe an appreciable population of slow-rebinding T-state molecules, that bind the photodissociated ligands at longer times [46–49].

The absorbance spectrum of the heme is strongly sensitive to ligation and can be exploited to monitor ligand conformational changes and ligand rebinding through the absorbance changes that occur concomitantly. Analysis of the time course as a function of ligand (CO) concentration, temperature, and other physiologically relevant solutes allows to determine rate constants for the various kinetic steps and their response to chemical-physical conditions.

The laser flash photolysis set-ups and the data analysis have been described previously [50–52]. Time evolution of CO-rebinding after nanosecond laser photolysis at 532 nm was measured by monitoring absorbance changes at 436 nm. The laser pulse repetition rate was kept at 1 Hz. Experiments were performed at 15 and 25°C, in the absence and presence of 2 mM ATP. CO- rebinding kinetics were also measured in the presence of 133 mM urea. The percentage of fast (R) and slow (T) rebinding in the bimolecular phase was estimated by normalising the amplitude of the corresponding exponential decay by the overall amplitude of the bimolecular phase. The bimolecular binding rate constant to R and T states is determined from the apparent rate (*k*_R_ = *1/τ*_R_; *k*_T_ = *1/τ*_T_) and the concentration of CO (*k*_on,R_ = *k*_R_ /[CO]; *k*_on,T_ = *k*_T_ /[CO]).

In laser flash photolysis experiments, concentrated stock solutions of purified *S*. *microcephalus* Hbs were diluted to about 30 μM [53] in a solution containing 0.1 M KCl, 0.1 M potassium phosphate, 1 mM EDTA, 2 mM ATP, at pH 6.5 or 7.4, in a cuvette fused with a gas-tight reservoir [53]. Before the experiments, the solutions were equilibrated with either pure CO (Rivoira Milan, Italy) or with a 10% CO/N_2_ mixture. Finally, sodium dithionite was anaerobically added to a final concentration of 2 mM to prevent Hb oxidation during the experiment.

### Autoxidation rate constants

In order to assess the stability of the oxygenated forms of *S*. *microcephalus* Hbs, their autoxidation rate (*k*_ox_) was measured at 25°C. For comparison, the autoxidation of HbA was measured under the same conditions. The proteins were first reduced to the ferrous form using an excess of sodium dithionite, which was then removed with a Sephadex G25 desalting column (GE Healthcare). They were then diluted to a final concentration of 30 μM (on heme basis) in a buffered solution containing 0.1 M Tris-HCl, 0.1 M KCl, 0.5 mM EDTA, at pH 7.45. Autoxidation was followed for 20 hours through time-resolved spectra (every 30 minutes), in a Cary 100 spectrophotometer (Varian, Inc) at 25°C. The reaction was monitored from 250 to 700 nm; the time courses were normalised using a spectrum collected immediately after exposure to oxygen and a spectrum obtained in the presence of sodium ferricyanide as references for the pure oxy- and met-forms, respectively. The initial phase of the reaction was fitted using a linear regression.

## Results

### Purification of Hbs, separation of globins and amino-acid sequence analysis

**Fig 1:**
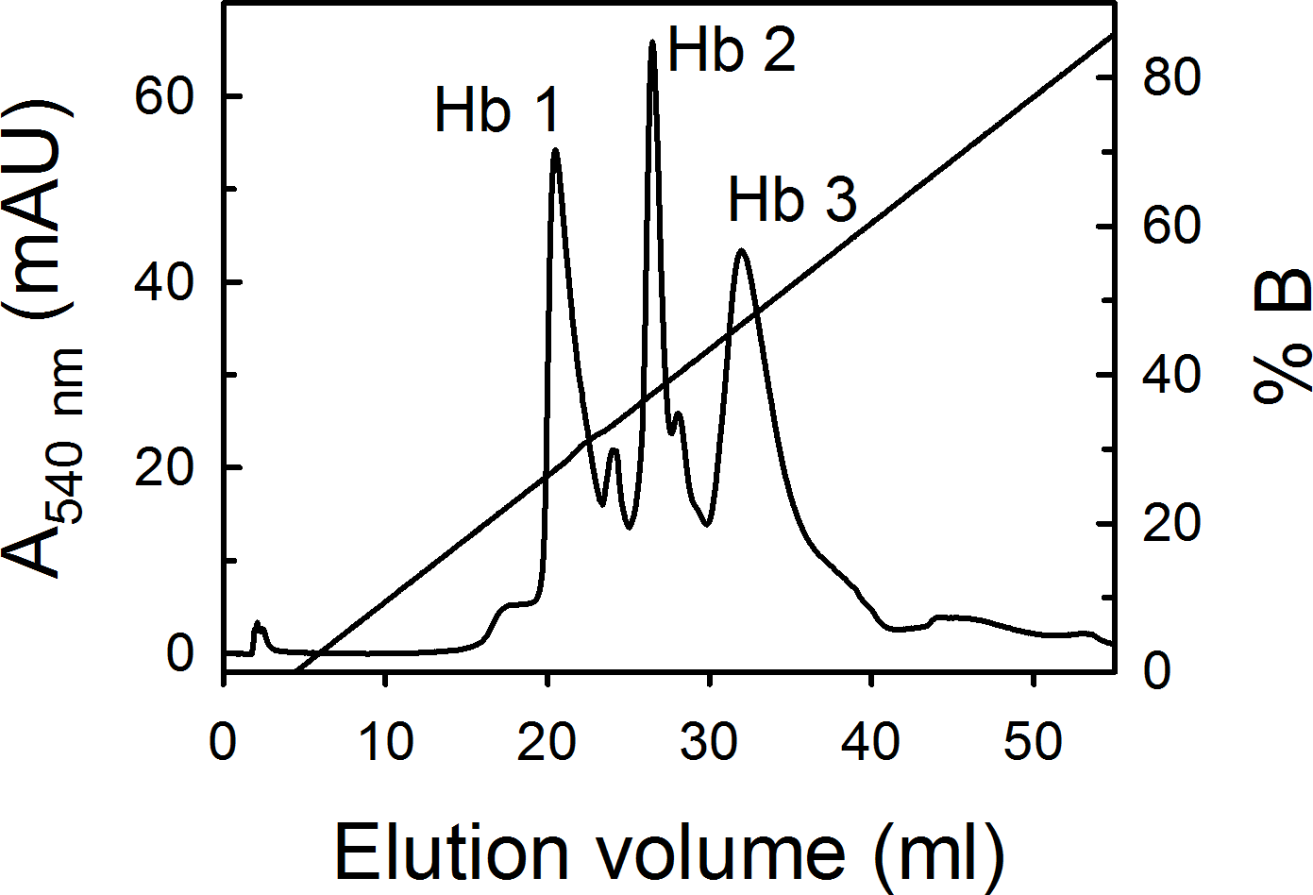
Ion-exchange chromatography of *S*. *microcephalus* hemolysate on a Mono Q-Tricorn column (1.0 × 10 cm). Elution was achieved with a linear gradient of 0 to 80 mM NaCl. Details are given in Materials and Methods.

Ion-exchange chromatography of the hemolysate showed three major and two minor Hbs (Fig 1), as confirmed by IEF on polyacrylamide gels (data not shown).

**Fig 2:**
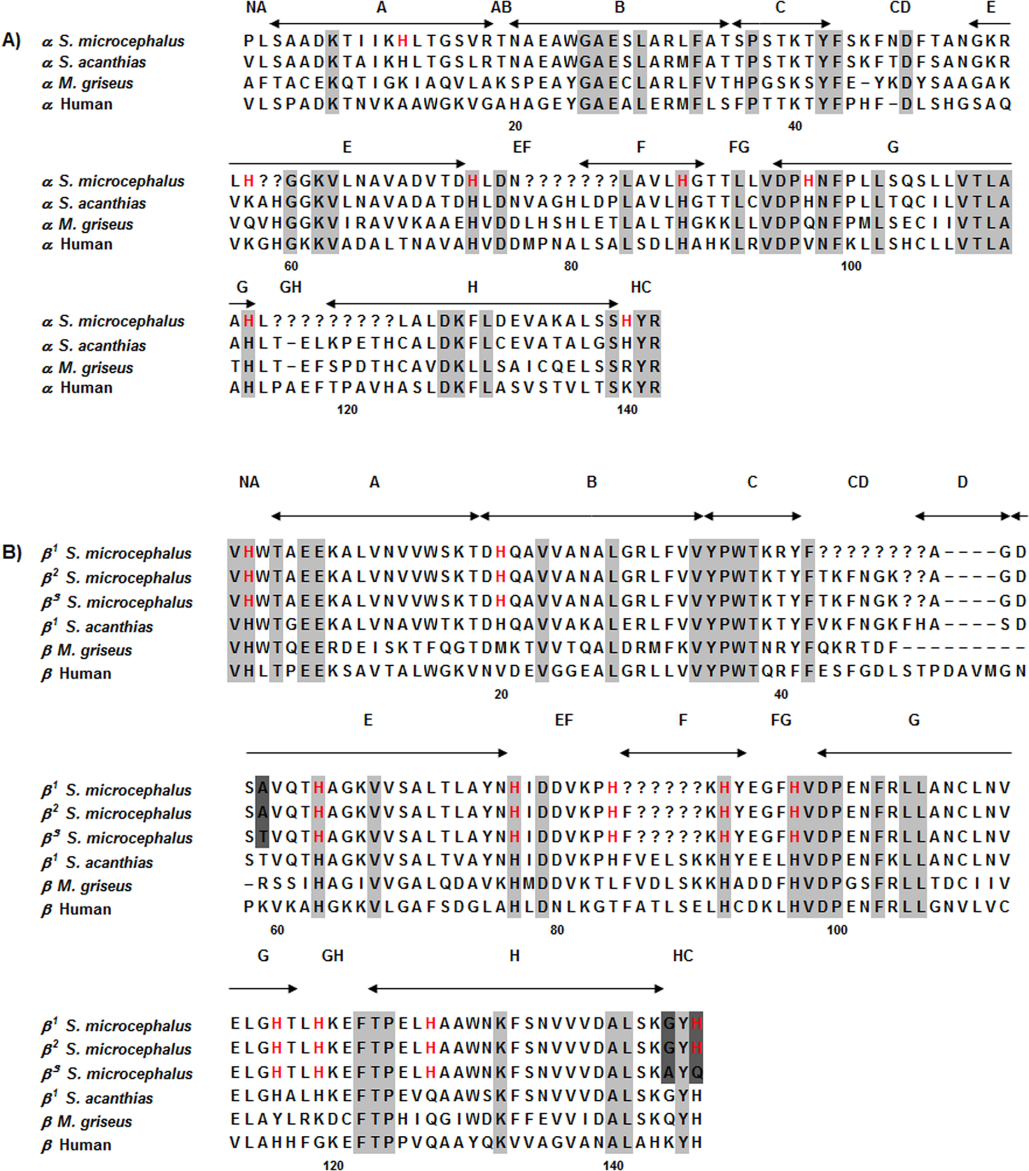
Amino-acid sequence alignment of the α (**A**) and β (**B**) chains of *S*. *microcephalus* Hbs with *S*. *acanthias*, *M*. *griseus* and *H*. *sapiens* α and β chains. Identical residues are in grey; different residues in the β globins of *S*. *microcephalus* are in dark grey. Histidyl residues of the α and β chains of *S*. *microcephalus* Hbs are in red. The chains were aligned using Clustal OMEGA; in the β chains of *S*. *microcephalus* the position of residues in CD and D have been manually aligned with the sequences of *M*. *griseus* [32] and *S*. *acanthias* [26] and HbA (accession numbers: P69905 for α chain and P68871 for β chain). The question mark indicates unsequenced regions; dashes indicate deletions.

The reverse-phase HPLC elution profiles indicated four major globins (data not shown), i.e. one α and three β chains (β^1^, β^2^ and β^3^), as established by amino-acid sequencing and mass spectrometry. Only few residues in the α and β chains, indicated by question marks in Fig 2, were not sequenced for technical reasons. The chain compositions of Hb 1, Hb 2 and Hb 3 are (αβ^1^)_2_, (αβ^2^)_2_ and (αβ^3^)_2_, respectively.

The α chain of *S*. *microcephalus* (accession number C0HJZ2) has 141 residues (Fig 2A). The β chains (accession numbers C0HJZ3, C0HJZ4, C0HJZ5) of *S*. *microcephalus* have 142 residues (Fig 2B), thus being four-residue shorter than the 146-residue *H*. *sapiens* β chains [54]. The sequence identity between β chains of *S*. *microcephalus* is very high, approaching 100%. From the alignment of the established sequences (Fig 2), β^1^ and β^2^ appear identical, but this view is not supported by the clear separation of the isoforms observed in the ion-exchange chromatography of the hemolysate (see Fig 1), as well as the three distinct β peaks observed in the HPLC elution profiles of the globins. However, the β sequences (especially β^1^) have portions in the CD corner and helix F that could not be sequenced, and one or more additional differences may be placed in these portions. We feel it is safe to hypothesise that the identity range is very high, with β^1^ and β^2^ perhaps differing by few residues and β^3^ being slightly less identical, because it certainly differs in at least 3 additional positions (Fig 2B). We cannot exclude that β^1^ and β^2^ are indeed identical, but may harbour differential oxidation of the cysteyl residues, also taking into account the longevity of the animal. Studies on the genome and single genes may clarify the issue.

### UV-Vis and resonance raman spectroscopy

The three isoforms of *S*. *microcephalus* display identical electronic absorption and RR spectra. Therefore, only the results obtained for Hb 3 are reported. S1 Fig (supplemental) shows the electronic absorption and RR spectra of the ferric form at pH 5.0, 7.6 and 10.6. The spectra are very similar to those reported for human HbA [55, 56]. At acidic pH, a pure six-coordinate (6c) aquo high-spin (HS) form is observed. This form remains predominant at pH 7.6, where weak bands assigned to the alkaline species grow in. At alkaline pH the spectra are typical of an OH^-^ ligated to the heme iron. As in HbA, the CT band at 603 nm together with the RR core size marker bands clearly indicate the presence of a hydroxo ligand bound to the heme iron (His-Fe-OH^-^), which exists in a spin-state equilibrium at room temperature, giving rise to a mixture of low- and high-spin species. Upon reduction, the protein gives rise to a 5cHS form, which fully binds either O_2_ or CO, as shown by the corresponding typical electronic absorption spectra (S2 Fig).

**Fig 3:**
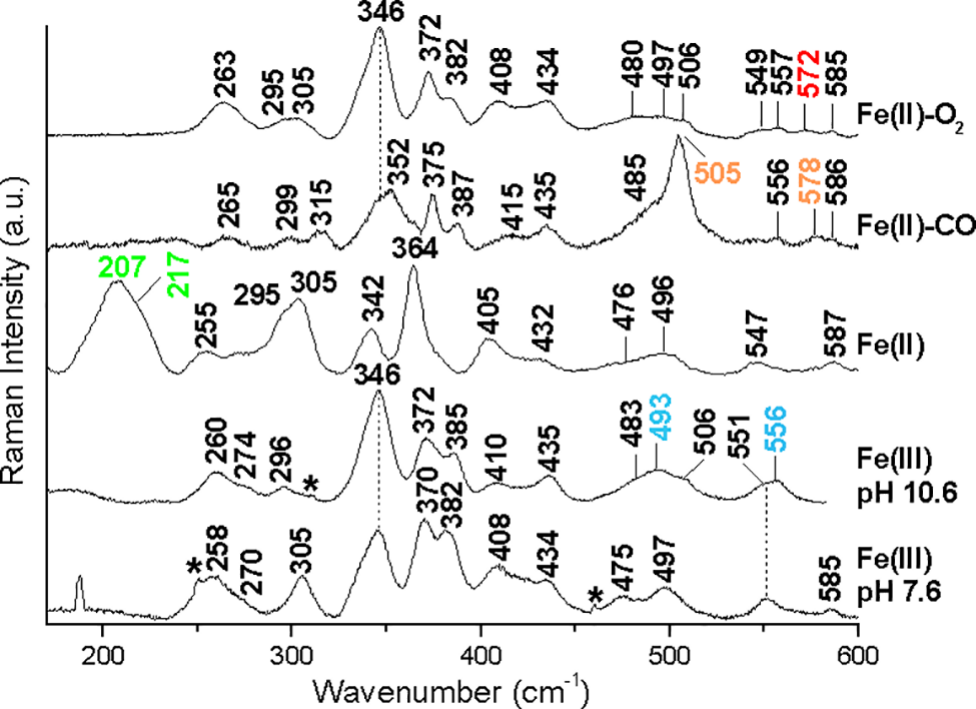
RR spectra in the low-frequency region of the ferric form at pH 7.6 and 10.6, ferrous form at pH 7.6, Fe(II)-CO and Fe(II)-O_2_ complexes of Hb 3. Experimental conditions: [Fe(III), pH 7.6]: excitation wavelength 406.7 nm, laser power at the sample 5 mW, average of 5 spectra with 25-min integration time; [Fe(III), pH 10.6]: excitation wavelength 413.1 nm, laser power at the sample 5 mW, average of 21 spectra with 105-min integration time; [Fe(II)]: excitation wavelength 441.6 nm, laser power at the sample 10 mW, average of 10 spectra with 50-min integration time; Fe(II)-CO]: excitation wavelength 413.1 nm, laser power at the sample 0.8 mW with cylindrical lens, average of 28 spectra with 140-min integration time; [Fe(II)-O_2_]: excitation wavelength 413.1 nm, laser power at the sample 5 mW with cylindrical lens, average of 56 spectra with 280-min integration time. The ν(Fe-OH), ν(Fe-Im), ν(Fe-C) and the δ(C-O), and the ν(Fe-O_2_) bands are shown in light blue, green, orange, and red respectively.The spectra have been shifted along the ordinate axis to allow better visualisation.

In order to obtain information on the bond strength and the interaction between the bound ligands and the proximal or the distal heme cavity sides, RR spectra in the low-frequency region have been obtained. Fig 3 shows the low-frequency RR spectra of the ferric form at both neutral and alkaline pH, and of the ferrous species before and after addition of O_2_ and CO. The comparison between the RR spectra of the ferric forms at pH 7.6 and 10.6, together with the experiments carried out at alkaline pH in D_2_O (S3 Fig), allowed us to identify the isotope sensitive ν(Fe-OH) stretching modes at 493 and 556 cm^-1^, for the HS and LS forms, respectively. Their frequencies are very similar to those observed in HbA [56].

Table 1 compares the RR frequencies of the various Fe-Ligand modes of Hbs from *S*. *microcephalus* with those of HbA. Moreover, similar to HbA [56], in the spectrum of Hb 3 in D_2_O, the ν(C_β_C_c_C_d_) propionate bending modes at 372 cm^-1^ in H_2_O up-shift by about 3–4 cm^-1^. Since in HbA the propionate groups are H-bonded to adjacent side chains [62], the frequency shift upon H/D exchange in Hb 3 could involve an alteration in the structure of the H-bonded propionate groups induced by the different strengths of H- and D-bonds.

**Table 1:** Comparison of the Fe-ligand mode frequencies of *S*. *microcephalus* Hbs with those of human HbA.

	ν(Fe-O_2_)	Fe-CO [Fe-^13^CO]			ν(Fe-OH) [Fe-OD]^a^		ν(Fe-Im)	
		ν(Fe-CO)	δ(Fe-CO)	ν(CO)	HS	LS		
HbA	568^c^	506^d^	578^d^	1951^e^	492 [479]	553 [544]	215 (T)^b^	
							203–207 (α)	217–220 (β)^b^^,^^f^
*S*. *microcephalus* Hb 1, Hb 2, Hb 3	572	505 [500]	578 [560]	1951 [1985]	493 [479]	556 [546]	207 (α)	217 (β)

^a^[56]

^b^[57]

^c^[58]

^d^[59]

^e^[60]

^f^[61]

Similar conclusions can be drawn for the ligands CO and O_2_. In the low-frequency RR spectra of the CO adduct, three isotope sensitive bands are identified (S4 Fig) at 505, 578 and 1951 cm^-1^, which are assigned to the ν(Fe-CO), δ(Fe-CO), and ν(CO) modes, respectively. These frequencies are almost identical to those of HbA [60]. The ν(Fe-O_2_) stretching mode of the oxy complex of Hb 3 was assigned to the band at 572 cm^-1^ on the basis of its pronounced intensity decrease at high laser power (data not shown). However, the intensity decrease is not solely due to photolysis of the O_2_ ligand, since upon increasing the laser power the formation of the ferric form is observed, possibly due to faster auto-oxidation of Hb 3 with respect to HbA (Table 2). The corresponding frequency for HbA is lower (568 cm^-1^) [63, 64]. The ferrous form is characteristic of a 5cHS (S2 Fig), and shows a strong band at 207 cm^-1^ with a shoulder at 217 cm^-1^ in the low-frequency RR spectra for excitation in the Soret band (λ_exc_ 441.6 nm), which is assigned to the ν(Fe-Im) stretching mode. In fact, as the ν(Fe-Im) stretching mode is observed only in the 5cHS ferrous heme proteins for excitation in resonance with the Soret, it decreases in the RR spectrum obtained with λ_exc_ 413.1 nm (S5 Fig) [65–67].

**Table 2:** Values of *P*_50_, *n*_*H*,_ *ΔH*, φ, k_off,R_ and k_ox_ of *S*. *microcephalus* Hbs (1 kcal = 4.184 kJ).

			*P*_*50*_ (Torr)		*n*_*H*_		*ΔH* (kcal·mol^-1^)		φ	k_off,R_ (s^-1^)	k_ox_ (min^-1^)
*S*. *microcephalus* Hbs	100mMKCl	2 mMATP	pH		pH		pH		pH	pH	pH
			**7.4**	6.7*	**7.4**	6.7*	**7.4**	6.7*	7.4–6.7*	**6.5**	**7.45**
**25°C**											
Hb 1	+	-	5.5	8.9	1.2	1.4	-7.9	-10.1	-0.2	13.1	2.2x10^-4^
	+	+	14.4	46.1	1.8	1.6	-0.7	-5.4	-0.6	10.5	
Hb 2	+	-	5.7	8.7	1.6	1.9	-8.9	-8.5	-0.2	7.1	3.1x10^-4^
	+	+	12.5	40.8	1.8	1.6	-2.1	-6.6	-0.6	6.6	
Hb 3	+	-	7.7	11.6	1.4	1.7	-8.4	-8.3	-0.2	35.1	3.9x10^-4^
	+	+	13.3	36.2	1.9	1.7	-0.9	-4.8	-0.5	33.3	
	100mMKCl	2 mM ATP	pH		pH				pH		
			**7.4**	**6.8**^§^	**7.4**	**6.8**^§^			7.4–6.8*		
**15°C**											
Hb 1	+	-	2.9	4.1	1.5	1.7			-0.2		
	+	+	11.6	28.2	2.1	1.3			-0.4		
Hb 2	+	-	2.8	4.4	1.6	2.0			-0.2		
	+	+	9.2	23.2	1.9	1.3			-0.4		
Hb 3	+	-	3.9	6.0	1.8	1.7			-0.2		
	+	+	10.6	22.9	2.0	1.7			-0.4		
**0°C**^†^			**7.8**	**7.4**	**6.7**							
Hb 1	+	-	0.9	1.0	1.2						
	+	+	6.2	8.1	12.6						
Hb 2	+	-	0.7	0.9	1.5						
	+	+	4.2	5.6	9.3						
Hb 3	+	-	1.0	1.3	2.0						
	+	+	5.8	7.3	10.8						

* pH 6.08 for Hb 3 in the presence of ATP

^§^ pH 6.01 for Hb 3 in the presence of ATP

^†^Extrapolated from Log*P*_*50*_ *versus* 1/T

Experimental errors are within 10%

*ΔH* values do not include the heat of O_2_ solubilisation

*P*_50_, O_2_ partial pressure at 50% of saturation; *n*, Hill coefficient; *ΔH*, enthalpy change; φ, Bohr coefficient, k_off,R_, dissociation constant referred to the R state; k_ox_, autoxidation rate

The ν(Fe-Im) stretching mode is observed at 215 cm^-1^ for deoxyHbA under physiological conditions [61, 64, 65, 68, 69]. Although this wavenumber is regarded as a marker of the T state, the observed ν(Fe-Im) RR band contains contributions from both the α and β subunits. Studies of valency-hybrid Hbs, including HbM Boston (αΜFe3+-βFe2+), HbM Milwaukee (αFe2+-βΜFe3+), α(Fe3+-CN)β(Fe2+-deoxy) and α(Fe2+-deoxy)β(Fe3+-CN), demonstrated a clear difference in the ν(Fe-Im) frequencies between the α and β subunits. It is now established that the ν(Fe-Im) modes of deoxyHb in the T state are located at 203−207 and 217−220 cm^-1^ in the α and βsubunits, respectively [61], consistent with the longer Fe-Im bond-length in the α compared to βsubunits in the crystal structure [70]. The two bands of Hb 3 have been assigned to the ν(Fe-Im) of the α (207 cm^-1^) and β (217 cm^-1^) subunits.

### O_2_-binding properties—Effect of ATP on O_2_ binding

The isoforms of *S*. *microcephalus* display very similar oxygenation properties and show similar Bohr effects (Table 2). The Bohr coefficient (φ = Δlog *P*_50_ ⁄ΔpH), which indicates the mean number of protons released upon heme oxygenation, was numerically higher in the presence of ATP (ranging from around -0.20 without ATP to -0.60 with ATP), with O_2_-linked dissociation of ~0.8 and ~2.0 protons in the absence and presence of ATP, respectively, *per* Hb tetramer. The enhancement by ATP was consistently high in all *S*. *microcephalus* Hbs, indicating that binding of negatively-charged ATP to the low-affinity conformation enhances H^+^ uptake. For comparison, the Bohr coefficient of human adult HbA in the presence of Cl^-^ is around -0.50 in the pH range of 7.0–7.4 [35, 37, 71]. *S*. *microcephalus* Hbs show cooperativity at all pH values investigated and at both temperatures (15 and 25°C), with *n*_*H*_ ranging from 1.2 to 2.0. Cooperativity increases in the presence of ATP. Moreover, the three isoforms show very similar organophosphate regulation.

*S*. *microcephalus* Hbs show *P*_50_ values similar to *M*. *griseus* Hb at pH 7.4 and 6.7 without ATP, under the same conditions [32]. At pH 7.4, 25°C, in the presence of ATP, *P*_*50*_ of *S*. *microcephalus* Hb 1, Hb 2 and Hb 3 was 14.4, 12.5 and 13.3 Torr, respectively, i.e. similar to *P*_*50*_ of *M*. *griseus* Hb (10.6 Torr) [32] and *D*. *akajei* Hb (11.80 Torr) [33] at pH 7.4, at 25°C. In contrast, at pH 6.7, 25°C, in the presence of ATP, *P*_*50*_ of *S*. *microcephalus* Hb 1, Hb 2 and Hb 3 (46.1, 40.8 and 36.2 Torr, respectively) (Table 2), was higher and fell between 20.1 Torr in *M*. *griseus* Hb and 61.6 Torr in *D*. *akajei* Hb at pH 6.5, with ATP [32, 33], indicating pH- and ATP-sensitive O_2_ affinity. *P*_*50*_ values of *S*. *microcephalus* Hbs at 0°C (Table 2), calculated by extrapolation from vant’Hoff plots [72], show the same behaviour of those at 15 and 25°C, in the absence and presence of ATP, indicating that the O_2_ affinity increases with decreasing temperatures up to the physiological conditions of cold waters. Moreover, *S*. *microcephalus* Hbs showed *n* values that increase in the presence of ATP but under all conditions are always below 1.9, whereas in *M*. *griseus* Hb *n*_*H*_ is always above 2.0, ranging from 2.2 to 2.6 [32]. The values of k_off,R_ for O_2_ are reported in Table 2. Those measured in Hb 1 and Hb 2 were almost identical, whereas Hb 3 exhibited a faster displacement kinetics under the same conditions. The modest differences are in agreement with the similar overall O_2_-binding properties observed in experiments at equilibrium (see Table 2). The presence of ATP did not affect k_off_ for O_2_—although a systematic decrease in apparent k_off_ was observed—suggesting that the 5-fold difference in O_2_ affinity at low pH is rather associated to k_on_, with possible contribution of k_off_ of the T state.

### Enthalpy change of Hb oxygenation

The regulation of the O_2_ affinity by temperature was investigated between 15 and 25°C (Table 2). *ΔH* (which includes the heat of O_2_ solubilisation–that in this case is excluded from the calculation to uniform our results with literature data—and the heats of proton and anion dissociation, i.e. processes linked to O_2_ binding), shows that the change of oxygenation is rather constant in the absence of ATP over the whole pH range explored (6.5–7.4), with the exception of Hb 1 for which it is more negative (i.e. oxygenation is more exothermic) at low pH. In the presence of ATP, the heat of oxygenation progressively decreases with decreasing pH, reflecting the endothermic contribution of the heterotropic effectors released upon O_2_ binding.

### Effect of urea on Hb oxygenation

The O_2_-binding parameters in the absence and presence of urea are summarised in Table 3. No important differences were observed at pH 7.4 and 6.5 (data not shown), either in terms of *P*_50_ or cooperativity.

**Table 3:** Influence of urea on O_2_-binding parameters of *S*. *microcephalus* Hbs in 0.1 M KCl, 0.1 M potassium phosphate, 1 mM EDTA, pH 7.4, 20°C, 2 mM ATP. Values of *P*_50_ are in Torr ± s.e.m. of two independent experiments.

	*P*_*50*_	*n*_*H*_	*P*_*50*_	*n*_*H*_
	no urea		133 mM urea	
Hb 1	13 ± 0.5	1.7 ± 0.10	12 ± 0.3	1.7 ± 0.07
Hb 2	1.2 ± 0.2	1.8 ± 0.06	9.9 ± 0.2	1.8 ± 0.06
Hb 3	12± 0.3	1.6 ± 0.08	11 ± 0.4	1.7 ± 0.10

### CO-rebinding kinetics

**Fig 4:**
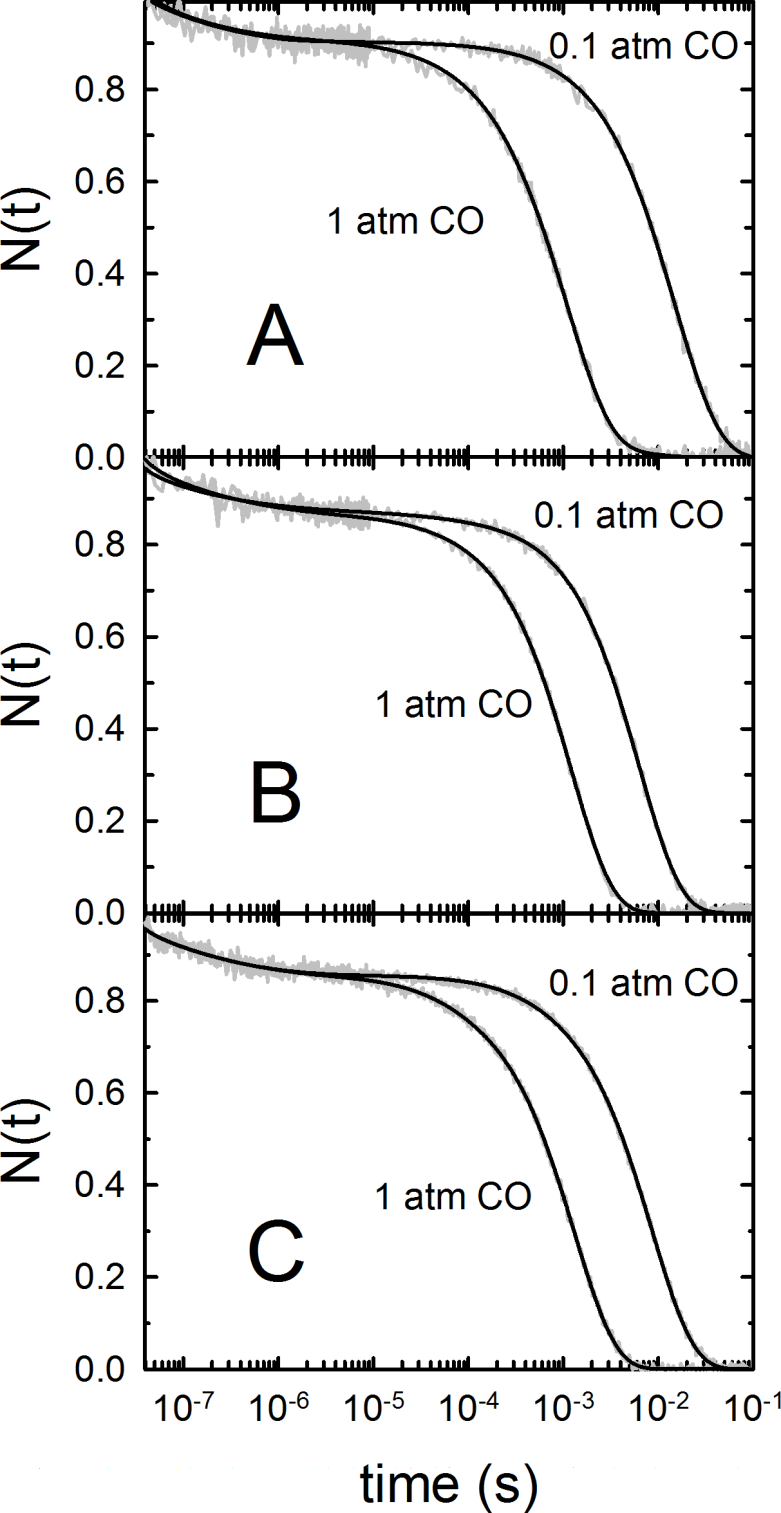
Transient absorbance at 436 nm after nanosecond photolysis of *S*. *microcephalus* Hb 1-CO (A), Hb 2-CO (B) and Hb 3-CO (C) at pH 7.4, 25°C, equilibrated with 0.1 and 1 atm CO (grey line). N(t) denotes the fraction of deoxy molecules (normalised absorption change). The black curves are the result of a fit with a sum of two stretched exponential and two exponential relaxations. The corresponding fractions of R and T forms and their binding rate constants are reported in Table 4.

**Fig 5:**
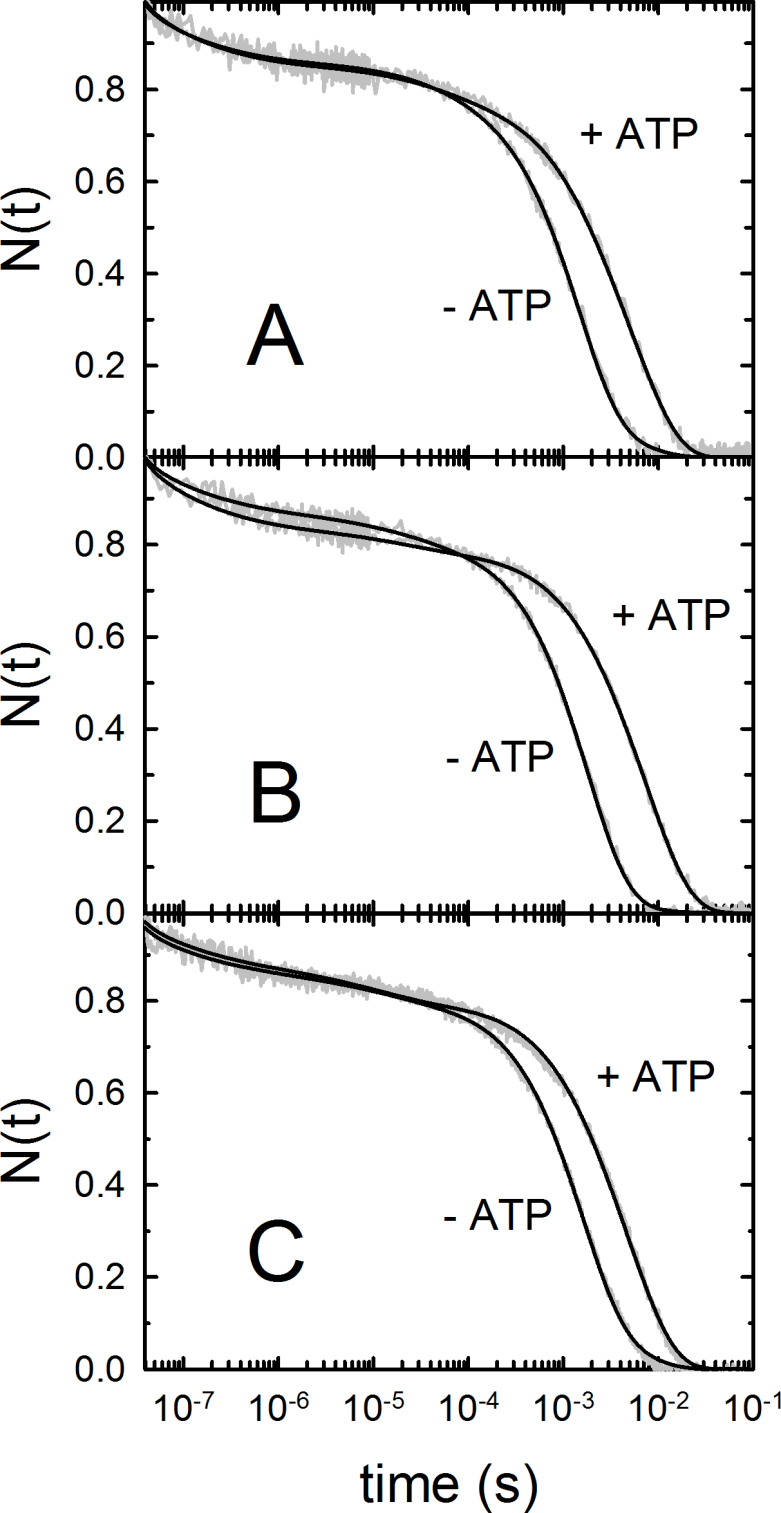
Transient absorbance at 436 nm after nanosecond photolysis of *S*. *microcephalus* Hb 1-CO (A), Hb 2-CO (B) and Hb 3-CO (C) in a solution containing 0.1 M KCl, 0.1 M Tris-HCl, 0.5 mM EDTA, 2 mM sodium dithionite, pH 7.4, 15°C, equilibrated with 1 atm CO in the presence and absence of 2 mM ATP (grey curves). The black curves are the result of a fit with a sum of two stretched exponential and two exponential relaxations.

Carbonylated Hb 1, Hb 2 and Hb 3 were photodissociated with a nanosecond laser pulse at 532 nm and the time evolution of CO recombination was followed by monitoring the absorbance changes at 436 nm. The resulting rebinding kinetics are shown in Fig 4 at two CO concentrations and under selected conditions (pH 7.4, T = 25°C, in the absence of ATP). The stretched exponential decays are a consequence of structural relaxation from the fast-rebinding form (R state) to the slow-rebinding form (T state), which is induced by photodissociation and overlaps in time to the rebinding reaction. The bimolecular phase is best described by a sum of two exponential decay functions, corresponding to rebinding to molecules in the R state and, at longer times, to molecules that have switched to the T state. In the presence of ATP, the bimolecular rebinding becomes slower (Fig 5).

The fractions of fast- and slow-rebinding states, whose values are obtained from the relative amplitudes of the bimolecular phases (Table 4), are ascribed to rebinding to R and T states, respectively. Consistent with the observed slower-rebinding kinetics, in all isoforms the fraction of T-state molecules (a measure of the extent of quaternary switch from R to T before the ligand is rebound) formed after photolysis increases upon addition of ATP. This finding is also in keeping with the observed decrease in affinity (Table 2).

**Table 4:** Rate constants and relative amplitudes for CO-rebinding kinetics following photolysis at 15°C and 25°C, pH 7.4 and 6.4, at 1 atm CO, from the global analysis of the kinetics reported in Fig 4 and Fig 5 for selected conditions.

			R (%)		T (%)		*k*_on,R_ (×10^5^ M^-1^s^-1^)		*k*_on,T_ (×10^5^ M^-1^s^-1^)	
	100 mM	2 mM	pH		pH		pH		pH	
	KCl	ATP	7.4	6.4	7.4	6.4	7.4	6.4	7.4	6.4
**25°C**										
Hb 1	+	-	84±4	10±8	16±2	0	9.4	4.2	2.4	2
	+	+	22±2	27±5	78±4	73±8	9.4	4.2	2.4	2
Hb 2	+	-	100±3	80±5	0	20±3	7	4.9	1.8	1.7
	+	+	12±2	26±2	88±3	74±4	7	4.9	1.8	1.7
Hb 3	+	-	92±3	68±2	8 ±1	32±2	7.3	6.5	2.8	1
	+	+	13±2	5 ±1	87±4	95±2	7.3	6.5	2.8	1
**15°C**										
Hb 1	+	-	90±4	90±1	10±2	10±2	5.9	2.3	1.3	1.3
	+	+	21±2	20±1	79±4	80±2	5.9	2.3	1.3	1.3
Hb 2	+	-	97±2	83±5	3 ±1	17±2	4.7	2.8	1.0	1.0
	+	+	13±1	25±2	87±3	75±4	4.7	2.8	1.0	1.0
Hb 3	+	-	85±2	96±2	15±1	4 ±1	5.5	4.2	1.5	0.5
	+	+	17±1	74±2	82±2	26±1	5.5	4.2	1.5	0.5

We have also checked the effect of urea on CO-rebinding kinetics in all three Hbs. The geminate phase was not affected by urea, whereas the second-order phase displayed a small but not negligible effect.

The effect of decreasing pH on the fraction of T-state molecules is less clear. While the affinity is invariably lower when pH is lowered to 6.4, the changes in the fraction of T-state rebinding do not always go toward the same direction (Table 2).

Table 4 also summarises the values of the bimolecular binding rate constants to R (*k*_on,R_) and T (*k*_on,T_) states. The ratio between these two parameters is definitely lower in *S*. *microcephalus* Hbs, being equal to 3.9 in Hb 1 and Hb 2, and to 2.6 in Hb 3. A similar ratio (4.5) was reported in the porbeagle shark (*Lamna nasus*) Hb [73]. ATP has negligible effects on the rate constants. Unlike ATP, pH was found to have some influence on rebinding rate constants. Lowering pH from 7.4 to 6.4 in Hb 1 leads to a 2-fold decrease in *k*_on,R_, while the effect becomes smaller in Hb 2 (1.4-fold) and Hb 3 (1.1-fold). Unlike the behaviour observed for the amplitudes of R and T rebinding (reflecting the extent of the R-to-T switch), the change in rate parallels the increase in *P*_50_ in all three isoforms. Only small differences were observed in k_on,T_ of Hb 1 and Hb 2, whereas a pronounced change was found only in Hb 3.

### Autoxidation kinetics

The autoxidation rate constants of *S*. *microcephalus* Hbs are also reported in Table 2. The values of k_ox_ (corresponding to the slope of the linear plot), obtained at pH 7.45 and 25°C, indicate that autoxidation of shark Hbs proceeds at a faster rate than HbA (1.3×10^−4^ min^-1^). The k_ox_ of Hb 3 (3.9×10^−4^ min^-1^) is slightly faster than those of Hb 1 and Hb 2, 2.2×10^−4^ and 3.1×10^−4^ min^-1^, respectively.

## Discussion

Shark Hb systems are characterised by the occurrence of Hb isoforms [74, 75]. As a whole, Hbs of *S*. *microcephalus* appear to be functionally quite similar as reflected by the high degree of sequence identity and similar ligand-binding properties, as also seen in other elasmobranchs [15]. This suggests the presence of Hb microheterogeneity in this species, i.e. without major functional specialization of Hb isoforms associated with migrations within wide latitudinal ranges. Unlike most mammals, teleosts often exhibit multiplicity of Hbs that display striking varieties among species from the structural and functional viewpoints [76, 77]. Multiplicity is usually interpreted as a sign of phylogenetic diversification and molecular adaptation, and generally is the result of gene-related heterogeneity and gene-duplication events [78, 79]. However, in cartilaginous fishes, there is no evidence for the radical functional differentiation often found in teleosts, and no information on the functional consequences of Hb multiplicity is available for sharks. The absence of functional heterogeneity in *S*. *microcephalus* Hbs could explain the general low tolerance of sharks to variation in O_2_ pressure, and their low capability to maintain constant O_2_-uptake rates as O_2_ tension falls [80, 81]. The fact that these Hbs have very similar properties also suggests that the shark may play regulatory control of O_2_ transport at other levels. Alternatively, these isoforms may protect against deleterious mutational changes in the globin genes, thus providing higher total Hb concentration in the erythrocyte, and increasing the expression rate of the genes [82].

As mentioned in the Results section, a higher Bohr effect in the presence of ATP was found in *S*. *microcephalus* Hbs in comparison to temperate sharks, thus suggesting evolution of molecular adaptations at the level of the Hb molecule. In *S*. *microcephalus* Hb 1 and Hb 2, HisβHC3 and LysαC5 are conserved; Hb 3 has Gln at position βHC3, whereas AspβFG1 is replaced by Glu, as in *M*. *griseus* Hb [32]. Valβ1 e Hisβ2, which contribute to the Bohr effect in adult human Hb (HbA) together with HisβHC3, forming salt bridges with Asp94βFG1 and LysαC5 [83], are also present in *S*. *microcephalus* (Fig 2B). These residues are also present in *S*. *acanthias* and *M*. *griseus* Hbs, which however show a smaller Bohr effect and weaker cooperativity [26, 32]. In contrast, *B*. *eatonii* and *R*. *hyperborea* Hbs lack pH and organophosphate regulation, consistent with substitutions of functionally important amino acid residues [17].

The globin chains of *S*. *microcephalus* have much higher degree of identity with those of *S*. *acanthias* than those of *M*. *griseus*. The sequence identity of *S*. *microcephalus* Hbs and human HbA is 50% for the α chain and between 44 and 47% for the β chains on the basis of the established sequence, with many replacements of functionally important residues. The *S*. *microcephalus* α chain differs from that of *M*. *griseus* [32] in showing a deletion in αCD3. In both species, an insertion is present in αCD5 with respect to adult human α chain HbA [26]. The highly conserved *C*-terminal sequence Tyr-Arg is found in all α globins examined.

In all *S*. *microcephalus* β chains, the four residues of helix D are missing. The lack of helix D in the β subunits is a feature which distinguishes cartilaginous from teleost Hbs. The Hbs of the polar skates *Bathyraja eatonii* and *Raja hyperborea*, of the sharks *S*. *acanthias* [26], *Heterodontus portusjacksoni* [84, 85] and *M*. *griseus* [32], and of the temperate skate *D*. *akajei* [33] do not display helix D in β subunits. Previous site-directed mutagenesis suggested that this deletion is a neutral modification, neither exerting large functional effect(s) on O_2_ binding nor affecting the assembly of cooperative tetramers [86]. The high number of histidyl residues in *S*. *microcephalus* Hb chains confirm the hypothesis that a high buffer capacity of the Hb was the ancestral state in all jawed vertebrates, similar to contemporary elasmobranchs [87].

All kinetic traces of the three Hbs of *S*. *microcephalus* show a faster phase (ns-μs) independent of CO concentration, which corresponds to the rebinding of CO within the protein matrix (geminate rebinding). This is followed by a slower phase (ms), dependent on the CO concentration, which is due to bimolecular rebinding of CO.

The geminate phase of *S*. *microcephalus* Hbs has a small amplitude and is best described by a sum of two stretched exponential decay functions, and is similar to the analogous phase observed in Hb 1 and Hb 2 of the sub-Antarctic teleosts *Eleginops maclovinus* Hb 1 [88] and *Dissostichus eleginoides* [89]. The bimolecular binding rate constants to R (*k*_on,R_) and T (*k*_on,T_) states are larger than those estimated in Hb 1 of *E*. *maclovinus*, *D*. *eleginoides* and *T*. *bernacchii* [88–90]. In these Hbs, the rate constant *k*_on,R_ is approximately one order of magnitude larger than *k*_on,T_. The ratio between these two parameters is definitely lower in *S*. *microcephalus* Hbs. A similar ratio (4.5) was reported in the porbeagle shark (*Lamna nasus*) Hb [73].

Under our experimental conditions, the R→T quaternary transition proceeds to a larger extent in the presence of ATP in all three isoforms, indicating faster kinetics for this process. The effect of ATP was also demonstrated in the Antarctic/sub-Antarctic teleosts *D*. *eleginoides* [89], *Trematomus bernacchii* [90] and *E*. *maclovinus* [88].

Urea does not alter the effect of ATP on the O_2_ affinity of *S*. *microcephalus* Hbs, as already reported in several ray species [91] and in the sandbar shark *Carcharhinus plumbeus* Nardo [44].

The global analysis of kinetics in the absence and in the presence of urea reveals that the population of the R state increases by 35%, thus indicating that urea influences the kinetic properties in an opposite way with respect to ATP; namely, in the presence of urea, the R→T quaternary transition proceeds to a lower extent before ligands are rebound (data not shown).

The low O_2_ affinity observed in *S*. *microcephalus* Hbs indicates some similarity with that of some Antarctic and sub-Antarctic teleosts of the suborder Notothenioidei [92] and of the Arctic fish *Arctogadus glacialis* [21], allowing us to speculate that the *P*_50_ values might be linked to the high O_2_ concentration in cold waters, the only property suggesting some adaptation to cold temperatures [93]. In fact, these observations highlight an important difference from temperate cartilaginous Hbs, and, in contrast, some similarity between a polar shark and cold-adapted teleosts thriving in both polar environments. Although amino-acid sequences and ligand binding did not reveal special features associated to cold environmental conditions, the decrease in O_2_ affinity evolved by the Hb isoforms of *S*. *microcephalus* may have adaptive implications.

Along the same lines, thermodynamic data show that the oxygenation-enthalpy change in *S*. *microcephalus* Hbs is lower than that of temperate fish Hbs and very similar to that of polar fish Hbs [21, 92]. The expression of Hbs with reduced *ΔH* seems a frequent evolutionary strategy of cold-adapted fish, resulting in improved O_2_ release to tissues at low temperatures [92]. The low temperature effect on O_2_ affinity in *S*. *microcephalus* Hbs thus suggests that O_2_ delivery may be facilitated by lower heat of oxygenation of Hb, as also reported in polar mammals [94].

The effect of ATP on the oxygenation of *S*. *microcephalus* Hbs differs from that of the Hb systems of the two polar cartilaginous ray species *B*. *eatonii* and *R*. *hyperborea* [17], which show absence of Bohr effect and organophosphate sensitivity. The inspection of the model of the ATP binding site in teleost Hbs [95, 96] indicates that Valβ1, Hisβ2, LysβEF6, and HisβH21, which bind 2,3-diphosphoglycerate (DPG) in human HbA, participate in ATP binding. These residues are conserved in *S*. *microcephalus* Hbs, with the exception of HisβH21, replaced by Lys, consistent with the allosteric effect of ATP on O_2_ affinity and Bohr effect. In *M*. *griseus* Hb, although the canonical binding site is not preserved, ATP works as allosteric effector by lowering the O_2_ affinity and stabilising the T-state structure [32]. The cooperating effect of urea and ATP on the O_2_ affinity has also been found in other elasmobranchs, e.g. the dogfish *S*. *acanthias* and the carpet shark *Cephaloscyllium isabella* [15, 97, 98].

In conclusion, our results indicate the absence of a significant structural and functional differentiation among the three Hb isoforms of the shark *S*. *microcephalus*. However, *in vivo* regulation of Hb function and O_2_ transport can be achieved *via* local blood pH shifts (Bohr effect) and changes in the concentration of physiological allosteric modulators within red blood cells, such as ATP. Additionally, differences in gene expression and in overall protein-synthesis may also contribute to regulate O_2_ transport in the short time.

We were also unable to identify patterns linked to cold adaptation to Arctic environment in the structure/function relationship of *S*. *microcephalus* Hbs. This suggests that physiological differences in O_2_ transport between polar and temperate sharks, both dispersed across wide latitude and temperature gradients, may be governed at the physiological plasticity level.

## Supporting information

S1 Fig Spectra of the ferric form of Hb 3 at pH 5.0 (red), 7.6 (black) and 10.6 (blue). **Left:** UV-Vis spectra, the 470–700 nm region is expanded 7- or 10-fold. **Right:** RR spectra in the high-frequency region. The intensity is normalised to that of the ν_4_ band. Experimental conditions: excitation wavelength 406.7 nm, laser power at the sample 5 mW, average of 4 spectra with 20-min integration time (pH 5.0) and average of 2 spectra with 10-min integration time (pH 7.6); excitation wavelength 413.1 nm, laser power at the sample 5 mW, average of 6 spectra with 30-min integration time (pH 10.6). The spectra have been shifted along the ordinate axis to allow better visualisation.
(PDF)

S2 Fig UV-Vis spectra of Hb 3. From the bottom: ferrous form, Fe(II)-CO and Fe(II)-O_2_ complexes. The 470–700-nm region is expanded 5-fold. The spectra have been shifted along the ordinate axis to allow better visualisation.
(PDF)

S3 Fig RR spectra in the low-frequency region of the ferric form of Hb 3 at alkaline pH in H_2_O (blue) and D_2_O (magenta). The spectra have been shifted along the ordinate axis to allow better visualisation. The intensity is normalised to that of the ν_4_ band. Experimental conditions: excitation wavelength 413.1 nm, laser power at the sample 5 mW, average of 21 spectra with 105-min integration time (pH 10.6) and average of 12 spectra with 60-min integration time (pH 11.0). The ν(Fe-OH) (bottom) and ν(Fe-OD) (top) stretching modes are shown in light blue.
(PDF)

S4 Fig RR spectra in the low- (left) and high- (right) frequency region the of the Fe(II)-^12^CO (bottom) and Fe(II)-^13^CO (top) complexes. The spectra have been shifted along the ordinate axis to allow better visualisation. Experimental conditions: excitation wavelength 413.1 nm, laser power at the sample 0.8 mW with cylindrical lens, average of 28 spectra with 140-min integration time (left, Fe(II)-^12^CO complex) and of 21 spectra with 105-min integration time (right, Fe(II)-^12^CO complex); excitation wavelength 413.1 nm, laser power at the sample 0.2 mW with cylindrical lens, average of 12 spectra with 60-min integration time (left, Fe(II)-^13^CO complex) and of 20 spectra with 100-min integration time (right, Fe(II)-^13^CO complex). The ν(Fe-C), δ(C-O) and ν(C-O) bands are shown in pink.
(PDF)

S5 Fig RR spectra in the low-frequency region the of the ferrous form of Hb 3 obtained with the 413.1 nm (bottom) and 441.6 nm (up) laser lines. The spectra have been shifted along the ordinate axis to allow better visualisation. Experimental conditions: excitation wavelength 413.1 nm, laser power at the sample 10 mW, average of 14 spectra with 70-min integration time (bottom); excitation wavelength 441.6 nm, laser power at the sample 10 mW, average of 10 spectra with 50-min integration time (up). The ν(Fe-Im) bands are shown in green.
(PDF)
