# Supplementary figures and images for: The Greenland shark Somniosus microcephalus—Hemoglobins and ligand-binding properties
Source: PLoS One. 2017 Oct 12;12(10):e0186181. doi: 10.1371/journal.pone.0186181 (PMC5638460; doi:10.1371/journal.pone.0186181)

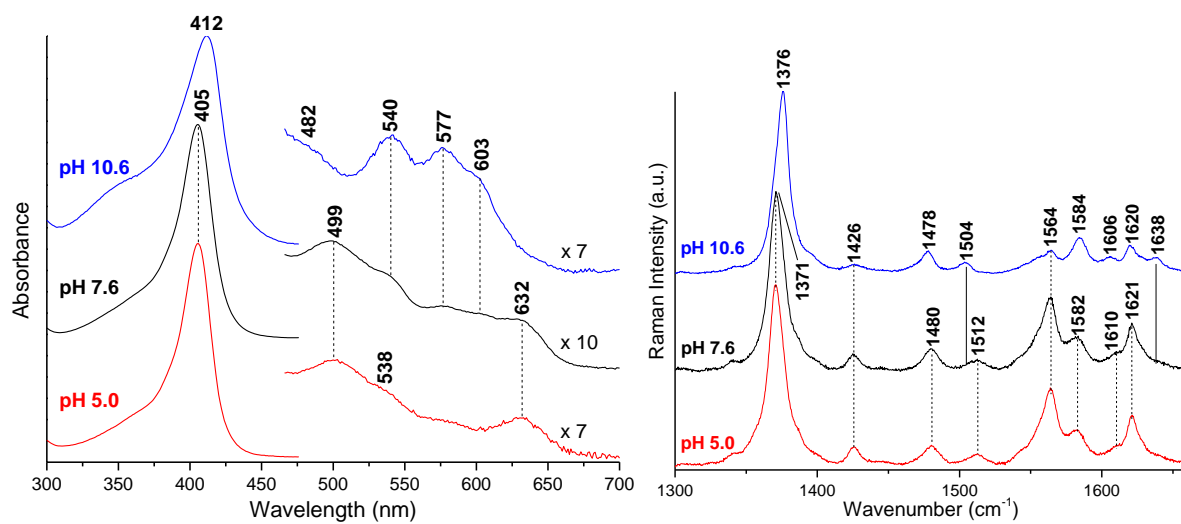

**S1 Fig.**

Supplement: S1 Fig — Left: UV-Vis spectra, the 470–700 nm region is expanded 7- or 10-fold. Right: RR spectra in the high-frequency region. The intensity is normalised to that of the ν4 band. Experimental conditions: excitation wavelength 406.7 nm, laser power at the sample 5 mW, average of 4 spectra with 20-min integration time (pH 5.0) and average of 2 spectra with 10-min integration time (pH 7.6); excitation wavelength 413.1 nm, laser power at the sample 5 mW, average of 6 spectra with 30-min integration time (pH 10.6). The spectra have been shifted along the ordinate axis to allow better visualisation. (PDF) [file pone.0186181.s001.pdf]

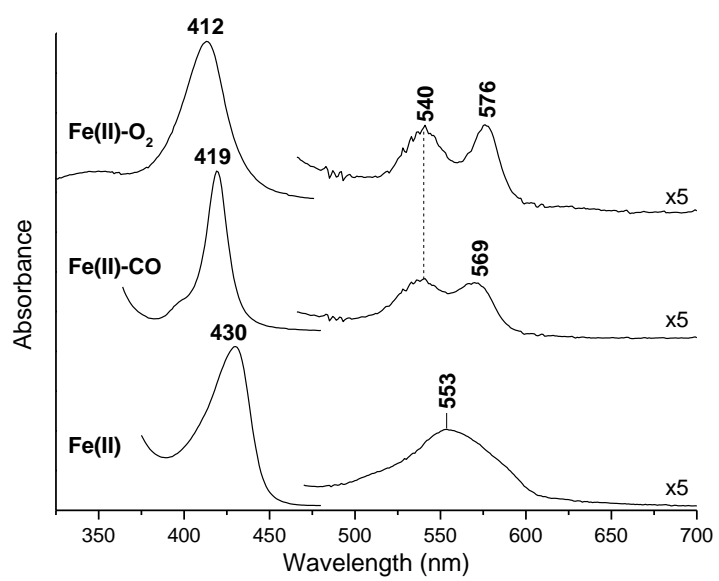

**S2 Fig.**

Supplement: S2 Fig — From the bottom: ferrous form, Fe(II)-CO and Fe(II)-O2 complexes. The 470–700-nm region is expanded 5-fold. The spectra have been shifted along the ordinate axis to allow better visualisation. (PDF) [file pone.0186181.s002.pdf]

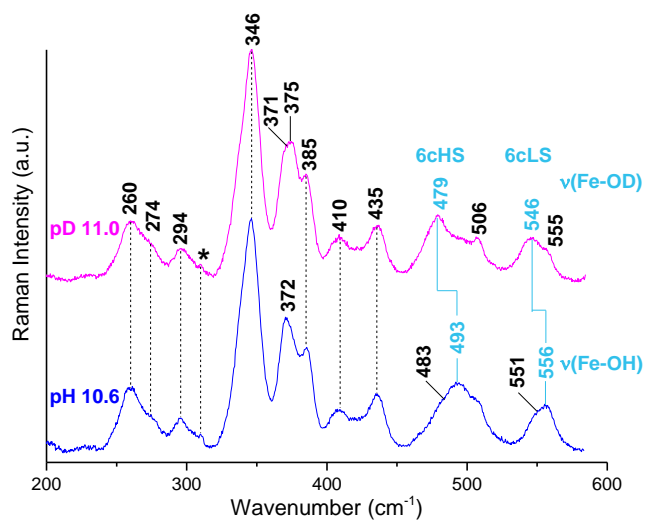

**S3 Fig.**

Supplement: S3 Fig — The spectra have been shifted along the ordinate axis to allow better visualisation. The intensity is normalised to that of the ν4 band. Experimental conditions: excitation wavelength 413.1 nm, laser power at the sample 5 mW, average of 21 spectra with 105-min integration time (pH 10.6) and average of 12 spectra with 60-min integration time (pH 11.0). The ν(Fe-OH) (bottom) and ν(Fe-OD) (top) stretching modes are shown in light blue. (PDF) [file pone.0186181.s003.pdf]

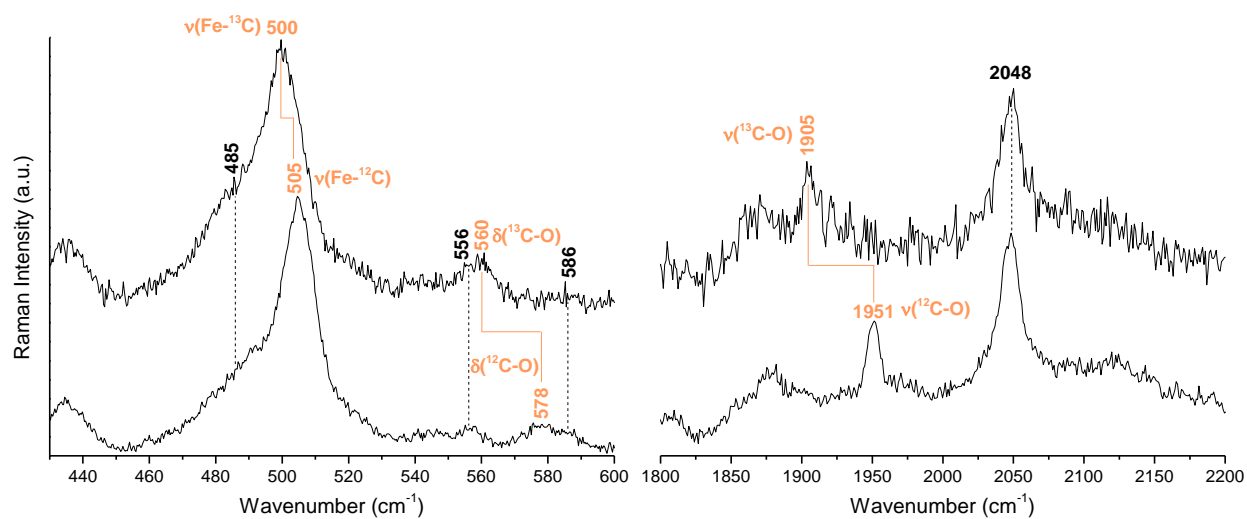

**S4 Fig.**

Supplement: S4 Fig — The spectra have been shifted along the ordinate axis to allow better visualisation. Experimental conditions: excitation wavelength 413.1 nm, laser power at the sample 0.8 mW with cylindrical lens, average of 28 spectra with 140-min integration time (left, Fe(II)-12CO complex) and of 21 spectra with 105-min integration time (right, Fe(II)-12CO complex); excitation wavelength 413.1 nm, laser power at the sample 0.2 mW with cylindrical lens, average of 12 spectra with 60-min integration time (left, Fe(II)-13CO complex) and of 20 spectra with 100-min integration time (right, Fe(II)-13CO complex). The ν(Fe-C), δ(C-O) and ν(C-O) bands are shown in pink. (PDF) [file pone.0186181.s004.pdf]

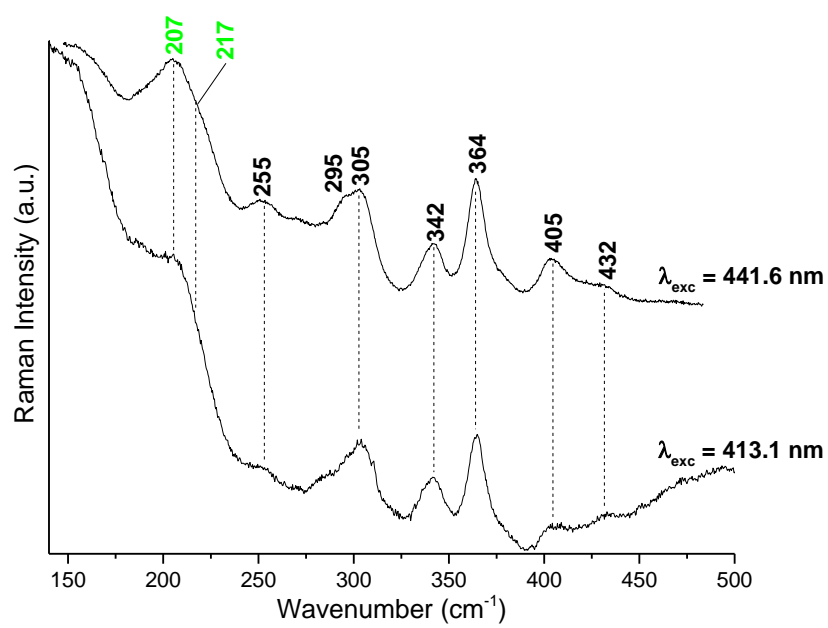

**S5 Fig.**

Supplement: S5 Fig — The spectra have been shifted along the ordinate axis to allow better visualisation. Experimental conditions: excitation wavelength 413.1 nm, laser power at the sample 10 mW, average of 14 spectra with 70-min integration time (bottom); excitation wavelength 441.6 nm, laser power at the sample 10 mW, average of 10 spectra with 50-min integration time (up). The ν(Fe-Im) bands are shown in green. (PDF) [file pone.0186181.s005.pdf]
